# Health-related quality of life in children and adolescents with paediatric acquired brain injury: Secondary data analysis from a randomised controlled trial

**DOI:** 10.1007/s11136-024-03838-2

**Published:** 2024-11-22

**Authors:** Hanna Lovise Sargénius, Torstein Baade Rø, Ruth Elizabeth Hypher, Anne Elisabeth Brandt, Stein Andersson, Torun Gangaune Finnanger, Kari Risnes, Jan Stubberud

**Affiliations:** 1 Department of Psychology, University of Oslo, Oslo, Norway; 2 Department of Clinical and Molecular Medicine, NTNU, Trondheim, Norway; 3 Children’s Clinic, St. Olav University Hospital, Trondheim, Norway; 4 Department of Clinical Neurosciences for Children, Oslo University Hospital, Oslo, Norway; 5 Psychosomatic Medicine and CL Psychiatry, Oslo University Hospital, Oslo, Norway; 6 Department of Research, Lovisenberg Diaconal Hospital, Oslo, Norway

**Keywords:** Paediatric, Acquired brain injury, HRQOL, EQ-5D-Y-3L, Executive function

## Abstract

**Purpose:**

To explore the characteristic quality of health profiles of children with paediatric acquired brain injury (pABI), and to investigate whether improvement in executive function (EF) following cognitive rehabilitation is associated with improvement in health-related quality of life (HRQOL).

**Method:**

A study of secondary endpoints in a blinded, parallel-randomised controlled trial with children (ages 10–17 years) with pABI and executive dysfunction. Data was obtained from 73 children-parent dyads. Explorative analyses were conducted comparing baseline with 8-week post-intervention, and 6-month follow-up data. Outcome measures included the EQ-5D-Y-3L health dimensions and the visual analogue scale (VAS).

**Results:**

At baseline and 6-month follow-up, mean (*SD*) VAS were 76.22 (17.98) and 79.49 (19.82) on the parent-report, and 77.19 (16.63) and 79.09 (17.91) on the self-report, respectively. Comparing children who improved EF to those who did not improve/worsened, no significant improvement was found for the VAS (parent-report) over time (BRIEF-BRI: *F* = 2.19, *p* = 0.12, BRIEF-MI: *F* = 2.23, *p* = 0.12) for either group. A significant main effect by group was found for BRIEF-MI (*F* = 4.02, *p* = 0*.049*), but no time*group interaction (*F* = 0.414, *p* = 0.662).

**Conclusion:**

The children and their parents reported only minor problems across EQ-5D-Y-3L dimensions and evaluated overall health as relatively good. Participants with a clinically significant change in the metacognitive aspect of EF had higher HRQOL. Cognitive interventions aiming to ameliorate deficits in EF in pABI may be beneficial to improve HRQOL.

**Supplementary Information:**

The online version contains supplementary material available at 10.1007/s11136-024-03838-2.

## Introduction

Any injury to the brain that occurs after birth, whether it be traumatic brain injuries (TBI) or non-traumatic injuries (e.g., brain tumour, cerebrovascular accidents, and infection) is categorised as a paediatric acquired brain injury (pABI). The consequences of pABI include long-term cognitive, behavioural and psychosocial problems and fatigue, that may restrict children’s opportunities for educational achievement, participation and quality of life [1–9]. However, the subjective impact of pABI on health-related quality of life (HRQOL) is still not fully recognised.

HRQOL is a multi-dimensional construct including physical, social, and psychological functioning, all relevant to overall health [10]. It is often approached in terms of how well individuals participate in a lifestyle typical for their age [11] and taking into consideration the impact their disease or illness might impose [12]. Individuals with pABI have been found to demonstrate similar levels of HRQOL to that of the general population [13, 14], but more often they seem to have much poorer scores on HRQOL measures compared to healthy peers or even other paediatric clinical populations [15–19]. Notably, findings on the associations between self-reported HRQOL and objective measures of health (e.g., injury severity indices and functional outcomes), are found to be weak, and rather more strongly related to reports of executive deficits, behavioural problems, fatigue, and limitations in participation [18, 20, 21]. Particularly a significant association between executive dysfunction and poor quality of life has been confirmed in children with pABI as well as other chronic paediatric conditions, such as epilepsy, developmental coordination disorder, and autism spectrum disorder [22–24].

Higher levels of HRQOL have been associated with better subjective executive function (EF) in children and adolescents with pABI [25]. From the perspective of clinicians, this could represent a new avenue for improving the lives of these children. Executive dysfunction and its effect on participation and overall quality of life is of great concern. Notably, the young brain is especially vulnerable to any disruption or insult that can cause deviations from the typical developmental trajectory of EF [9, 26]. EF is a concept that refers to a set of higher-level controlled cognitive processes, such as inhibition, mental flexibility, and working memory. These functions subtend appropriate goal-directed behaviour and are therefore critical in managing the multiple and complex tasks of daily life [27]. To date, evidence-based interventions to improve EF as well as overall health in children and youth in the chronic phase of pABI is limited compared to the adult ABI literature [28, 29].

In 2018 our group conducted a multicentre double-blinded, parallel group randomised controlled trial (RCT) on children in the chronic phase of pABI (> 12 months since injury/illness or completion of cancer therapy), with parent-reported executive complaints [30, 31]. Here, we investigated the efficacy of a paediatric adaptation of Goal Management Training (pGMT) relative to a psychoeducational program (paediatric Brain Health Workshop, pBHW). An integral part of the RCT was to explore the experiences made by the children and their families throughout the intervention as the new skills, strategies, and knowledge were absorbed and consolidated, and how they perceived the effectiveness of the intervention. After all, the perspective of the patient—in this case the child and his/her caregivers—could be the most potent indicator of the quality of treatment outcome [32]. Therefore, self- and proxy-reports were conducted at all time-points. In the questionnaire package provided, the EuroQol 5D children and adolescent versions (EQ-5D-Y-3L) [33] were included as a quality indicator of the intervention. The EQ-5D-Y-3L is part of the EQ-5D family of instruments and it is recognised being a relatively simple, yet a good tool for self-assessment of HRQOL in healthy populations as well as children and adolescents with disabilities [34, 35]. The various EQ-5D instruments can in many instances be more favourable compared to the more disease-specific measures within rehabilitation, as they in addition to low respondent burden include a health profile with broad relevance across health problems and/or diagnoses [32].

The present study presents the subjective experiences made by the participants throughout the above-mentioned RCT in regard to self-reported health. Specifically, the aims are to explore the characteristic health profiles and patterns of HRQOL across the five health states of EQ-5D-Y-3L for children and adolescents who undergo cognitive rehabilitation as perceived by both the youths and the parents, and the degree of agreement. Furthermore, we aim to investigate whether improvement in EF following cognitive rehabilitation is linked to perceived improvement in HRQOL. Considering the promising results of improved EF in pABI following intervention [31], it could be expected that HRQOL may improve equally. However, as the improvement of cognitive function seems to be mainly involving the metacognitive facet of EF and not the behavioural, whether HRQOL shows distinct patterns in relation to the two accordingly is undecided.

## Methods

### Study setting and design

The data used in the present study has been collected at three time points in tandem with a multicentre double-blinded, parallel group RCT. We refer to the previously published study protocol [30] for detailed information on study design, inclusion/exclusion criteria, and intervention contents and structure. In essence, participants were randomised (computer-algorithm) to either group-based pGMT (*n* = 38) or pBHW (*n* = 38). The active control (pBHW) was tailored to keep non-specific factors constant [31]. The two treatment groups each comprised seven modules of 2 h duration led by experienced clinical neuropsychologists. The modules had to be completed in a consecutive order. Following the fourth session, the children received additional external cuing by text messages. A combination of both in-session practice and between-sessions exercises were applied. Participants in both treatment groups were further divided into smaller groups of four children each. The groups provided the participants with a safe space for discussions concerning own injury and personal life experiences. The parents were throughout the process offered group counselling and support for applying the various techniques into everyday activities and asked to consecutively review the content of the intervention [36].

The RCT was approved by the Regional Committee for Medical and Health Research Ethics (2017/772), Norway, and conducted in accordance with principles of Good Clinical Practice, the Helsinki Declaration and the standards for Ethical Research Involving Children (ChildWatch International and UNICEF).

#### Sample

Eligible participants for the RCT were located based on hospital discharge diagnosis and record information at three hospital sites in Norway and were contacted by written invitation. For participants < 16 years, written informed consent was provided from their primary caregivers, and for participants > 16 years both the participant and their caregivers gave their consent. Seventy-three (96%) children and adolescents, and their parents, completed the allocated intervention. At 6-month follow-up, two participants failed to attend due to cancer recurrence, resulting in 93% attendance. (See supplementary S1 Figure for flow chart on recruitment.)

A summary of demographic, medical, and cognitive characteristics is provided in Table 1. A majority of the sample were girls (57.5%). Brain tumour was the dominant cause of pABI (*n* = 28), infection inflammation being the second largest group (*n* = 17), followed by TBI (*n* = 16). The mean time since injury was 4.8 (2.8) years, and mean age at inclusion in the study was 13.5 (2.3) years. Although reported EF problems were an inclusion criterion, the pABI group displayed cognitive functioning within the normal range relative to normative data on neuropsychological tests. The median global outcome on the paediatric version of the Glasgow Outcome Scale Extended (GOSE Peds) was Level 6 (range 3–8) corresponding to upper moderate disability level.

**Table 1:** Sample descriptives

	N = 73
Gender (*n*) Boys/girls	31/42
Age (years) at intervention *M*(*SD*)	13.48 (2.29)
10–13 years (*n*)	37
14–17 years (*n*)	36
*Injury characteristics*	
Age (years) at injury *M* *(SD)*	8.12 (3.66)
Time (years) since injury *M* *(SD)*	4.85 (2.77)
Primary injury aetiology (*n*)	
Traumatic brain injury	16
Brain tumour	28
Cerebrovascular accidents	5
Infection/inflammation	17
Hypoxia/Anoxia	7
GOSE Peds *M*(*SD*)	5.65 (1.45)
Intellectual abilities FSIQ *M*(*SD*)	92.49 (13.53)
BRIEF-BRI (% below clinically elevated)	79.5
BRIEF-MI (% below clinically elevated)	68.1

GOSE Peds: Glasgow Outcome Scale Extended Paediatric. Intellectual abilities measured with Wechsler Intelligence Scale for Children—5th Edition (WISC-V). FSIQ: Full scale IQ, *n* = 69. Behaviour Rating Inventory of Executive Function, Behavioural Regulation Index (BRI) and Metacognition Index (MI): *T* scores > 65 indicate scores within the clinically significant range for dysfunction

#### Main findings of the RCT

The co-primary outcomes 6 months post-intervention were the Behavioural Regulation Index (BRI) and Metacognition Index (MI) of the questionnaire Behaviour Rating Inventory of Executive Function (BRIEF) parent-report [37]. The BRIEF assesses parent-reported daily life EF, with the BRI reflecting the behavioural regulation facet to EF and the MI representing the metacognitive. Consistent with the BRIEF manual to indicate clinical significance, 1.5 SD above the mean (i.e., T ≥ 65) was used as cutoff.

The study found no significant difference between the two intervention groups for the primary outcomes (changes in parent-reported BRIEF raw scores from baseline to 6 months follow-up). Despite no difference in effect for the two interventions was demonstrated, the results indicated a significant decrease in parent-reported executive dysfunction for both intervention groups. Neither self- nor teacher-reports for BRIEF-BRI and BRIEF-MI demonstrated any differences in effect, further supporting the primary results [31].

### Measures

#### Study outcome

The present study used the three-level EQ-5D-Y-3L parent- and self-report [38] to measure overall HRQOL. The EQ-5D-Y-3L is a standardised instrument developed by the EuroQol group to assess child and adolescent HRQOL by five dimensions of mobility, self-care, usual activities, pain/discomfort, and anxiety/depression with a three-level descriptive system of “no problems”, “some problems”, and “a lot of problems” for each of the dimensions. Responses are coded thereafter as single-digit numbers (1, 2, or 3), and the digits for the five dimensions provide the respondent’s health state (i.e., 11111 representing no problems in either of the dimensions, and 33333 representing extreme problems in all five dimensions). Additionally, a visual analogue scale (VAS) gathers a rating (“we would like to know how good or bad your health is TODAY”) of the current overall health state as perceived by the individual ranging from 0 (the worst health status) to 100 (the best health status). The proxy-version has the same characteristics as the self-report version. Due to low frequency across some and extreme problems on the health dimensions, a dichotomised variable was created: one category of having no problems, and a second including both some problems and a lot of problems responses. Only parent-reports have been explored in the main LMM analyses. Self-reports are used primarily in the initial and/or in supplementary analysis. Cronbach’s α ranged from acceptable to good for the parent-reports (T1 = 0.70, T2 = 0.74, T3 = 0.84), and low to acceptable for the self-reports (T1 = 0.72, T2 = 0.68, T3 = 0.76).

#### Supplementary descriptor measures

The following supplementary descriptor measures were included in this study: (i) Baseline assessment of general intellectual abilities (i.e., full-scale IQ) assessed by the Wechsler Intelligence Scale for Children-Fifth edition, WISC-V [39]; (ii) The Glasgow Outcome Scale Extended, paediatric version, GOSE [40]; (iii) The Behaviour Rating Inventory of Executive Function (BRIEF) parent-report [37]. For a complete list of all measures applied in the RCT with interpretation, see previously published study protocol [30].

### Statistical analysis

All analyses were conducted using SPSS 29.0 and MedCalc 22.020, and conducted on pooled data across intervention groups after no group differences on the primary outcome measures in the RCT was found [31]. Frequencies and percentages of responders on the categorical measures (e.g., having no problems vs. having some problems/having severe problems) on the EQ-5D-Y-3L dimensions), and means and standard deviations (*SD*) of raw scores on the continuous variables (e.g., EQ-5D-Y-3L VAS) are reported. The analysis of the EQ-5D-Y-3L examined three time points: baseline (T1), 8 weeks post-intervention (T2), and 6-month follow-up (T3). Comparison with healthy population norms and application of the EQ-5D-Y-3L Index score was not conducted as there is yet not a social value set available for the EQ-5D-Y-3L for Norway.

Fisher’s exact tests for the five dimensions were conducted at all time-points for both parent- and self-reports, to examine the relationship between the response of reported problems and sex, as well as age-group. Mann Whitney U tests were used to investigate group differences on the VAS for the same grouping variables (age-group and sex), in addition to parents vs. youths at 6-month follow-up. Child-parent agreement was estimated using Cohen’s Kappa for the EQ-5D-Y-3L dimensions, and Lin’s Concordance Correlation Coefficient (CCC) for the VAS. Furthermore, to obtain a more detailed overview of the type of child-parent agreement of severity on the five health dimensions, three categories labelled “child = parent”, “child > parent”, and “child < parent” was computed for descriptive measure [41].

**Fig. 1:**
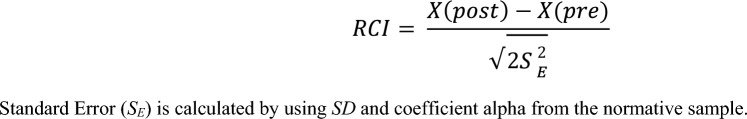
Calculation of the Reliable Clinical Change Index

A Reliable Change Index (RCI) was calculated for each participant based on parent-reported BRIEF index scores, BRI and MI. The RCIs were calculated according to the formula below (Fig. 1), using each participant’s pre-test and post-test scores, and the standard deviations (*SD*) and coefficient alpha (*r*_nn_) from the normative sample for the scale [42]. A series of Spearman correlations was applied to examine the relationships between the parent-reported VAS and the RCIs calculated from the raw scores of BRI and MI. A significance level of < 0.05 was employed in all analyses.

From the calculated RCIs, a new variable was created and coded into “negative clinical change” (including “no clinical change”) and “positive clinical change”. Potential within and between group differences of reported VAS on the EQ-5D-Y-3L parent-reports for the two main clinical change groups were explored by linear mixed-modelling (LMM) using unstructured and restricted maximum likelihood (REML) for estimation. The model included clinical change-group, time (baseline, post-intervention (T2), and 6 months follow-up (T3), and the interaction of time and clinical change-group as fixed factors. Significance was set to < 0.05. Bonferroni corrections for multiple comparisons were only applied for the fixed factors in the LMM individually. Sex and age-group was added as covariates in the analysis to adjust for potential influences on significant clinical change group*time interactions.

## Results

### Description of EQ-5D-Y-3L health profiles and domain severity

At baseline (T1), 29 (parent-report) and 27 (self-report) unique health profiles emerged, with a slight reduction at 6-month follow-up (T3) with 22 (parent) and 19 (self) unique health profiles respectively. The health profile of 11111 was for both the parent- and self-reports the most common profile at T1 (Parent: 23.3%, Self: 24.7%, and T3 (Parent: 31.5%, Self: 30.1%). Only one 33333-profile (parent) and one 33332-profile (self) emerged, which was the case for both T1 and T3. The dimensions of “Mobility” and “Looking after myself” were the least affected dimensions on both the parent- and self-report at all time-points (Table 2). The most affected dimensions for both parent- and self-reports were “Having pain or discomfort”, and “Feeling worried, sad, or unhappy”. (See supplementary S1 Table for an overview of the distribution of the children and parent category responses for severity on the five EQ-5D-Y-3L health dimensions.)

**Table 2:** Child-parent agreement on the health dimensions

	T1			T2			T3		
	Kappa	p		Kappa	p		Kappa	p	
Mobility	0.591	< 0.001	Moderate	0.595	< 0.001	Moderate	0.613	< 001	Good
Self-care	0.497	< 0.001	Moderate	0.562	< 0.001	Moderate	0.608	< 001	Good
Usual activities	0.470	< 0.001	Moderate	0.564	< 0.001	Moderate	0.534	< 001	Moderate
Pain or discomfort	0.338	= 0.004	Fair	0.609	< 0.001	Good	0.558	< 001	Moderate
Worry	0.413	< 0.001	Moderate	0.572	< 0.001	Moderate	0.476	< 001	Moderate

Due to low frequency across some and extreme problems, a binary measure was created: one category of having no problems, and a second including both some problems and extreme problems responses. Altman’s scale interpretation: less than or equal to 0.2: poor, between 0.21 and 0.4: fair, between 0.41 and 0.6: moderate, between 0.61 and 0.8: good, between 0.81 and 1: very good

Differences in proportion of reported problems among boys and girls were to some degree found in both the parent- and the youths self-reports (data not included), although trends were not consistently throughout T1 to T3. “Mobility” and “Self-care” were most problematic for boys according to their parents. In contrast, girls were by their parents reported to have more problems of being worried. The only dimension revealing a close to or significant proportional difference as reported by the youths themselves, was the dimension of Worry, with higher numbers of girls. No associations between the differentiating age-groups (10–13 and 14–17 years) and health dimensions were found for either of the parent- and youth self-reports.

#### Child-parent agreement on the EQ-5D-Y-3L dimensions

For the five EQ-5D-Y-3L dimensions, when using the dichotomisation of having no problems vs. some and extreme problems responses, the child-parent dyads revealed mostly fair to good agreement, with the dimension of “Pain and discomfort” at T1 as the weakest (Table 2). Parent–child agreement improved or remained relatively stable on the dimensions of “Mobility”, “Self-care”, and “Participation in usual activities” from T1 to T3. Agreement on the dimensions of Pain and that of Worry, both improved at T2, but declined at T3.

**Fig. 2:**
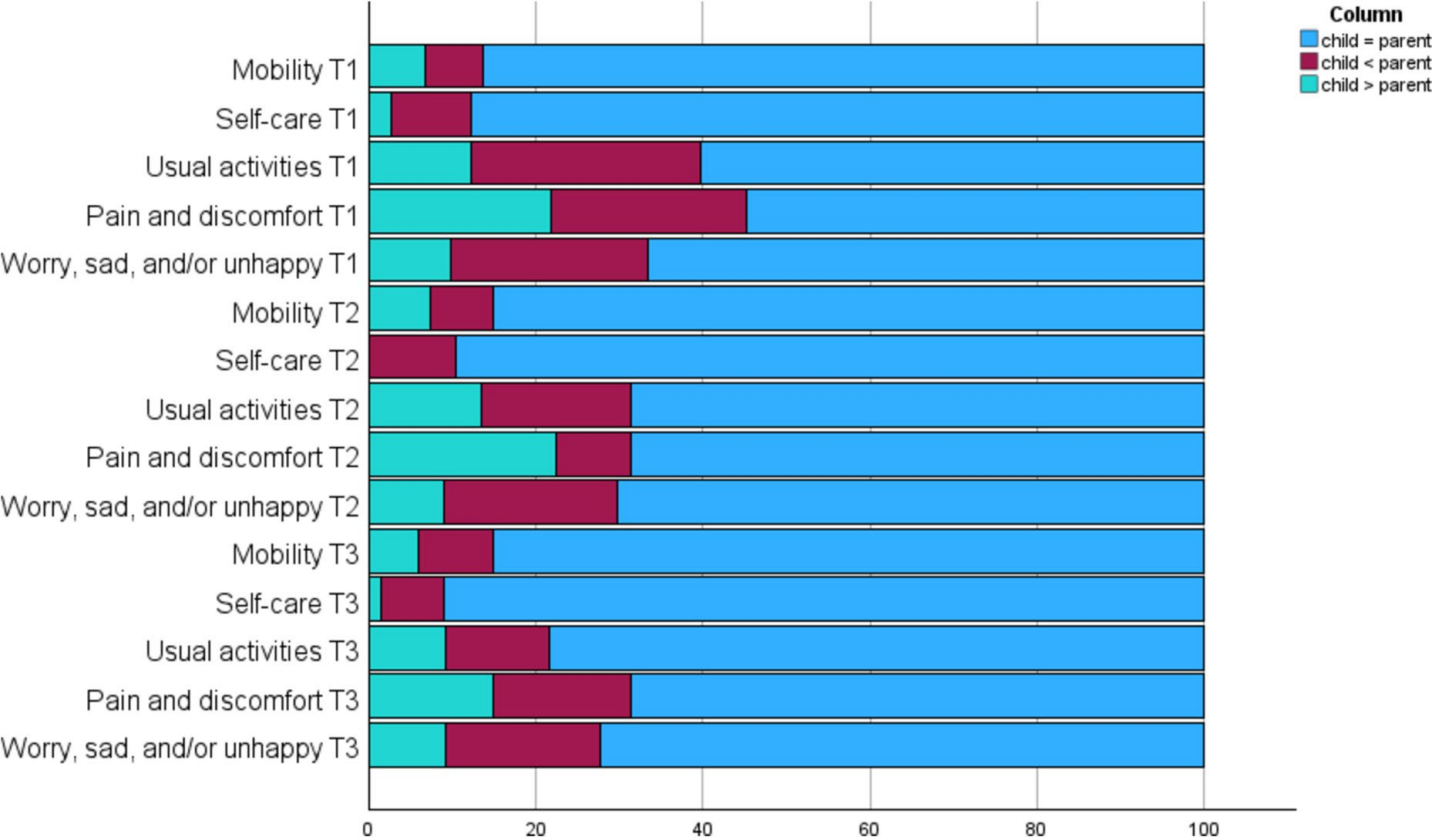
Distribution of response categories of severity for the health dimensions reported in percent. T1 = 73 complete dyad responses on all dimensions except “worry, sad, and/or unhappy” with 72 complete. T2 = 67 complete dyad responses on all dimensions. T3 = 67 complete dyad responses on “mobility”, “self-care”, and “pain and discomfort”, 64 complete dyad responses on “activities”, and 65 complete dyad responses on “worry, sad and/or unhappy”

Figure 2 illustrates the distribution of agreement and disagreement types between children and parents across the five health dimensions. From T1 to T3, there is an overall increase in the frequency with which children and parents selected the same response category for severity (child = parent). The dimension “looking after myself” had the highest frequency of agreement, while the dimension “feeling pain or discomfort” consistently had the lowest frequency at all time points. For the two categories of disagreement (child < parent, child > parent), across all observations, in only two (separate) cases and on two different dimensions a child-parent dyad did not chose adjacent response categories.

### EQ-5D-Y-3L VAS

On the question of how the participants perceived their overall health as being good/bad as rated along a scale of 0–100, only minor fluctuations were found throughout the intervention. The mean VAS (see Table 3) for both parent- and self-reports increased from T1 to T3, with a slight reduction at T2 on the self-reports, with the younger children having higher scores relative to the older children/adolescents as reported by parents. No significant group differences in VAS emerged between boys/girls or younger/older children, however. Parent–child agreement was moderate to good.

**Table 3:** VAS descriptives and child-parent reported agreement T1–T3

*M* (SD)	T1	T2	T3
Overall VAS	*N* = 73 (*n* = 73/73)	*N* = 66 (*n* = 68/67)	*N* = 67 (*n* = 69/67)
Parent-report	76.22 (17.98)	77.35 (20.23)	79.49 (19.82)
Self-report	77.19 (16.63)	75.87 (21.32)	79.09 (17.91)
Agreement CCC [95% CI]	0.47 [0.28–0.63]	0.64 [0.48–0.77]	0.65 [0.49–0.77]

VAS by age-group			
Age-group 10–13	*N* = 37 (*n* = 37/37)	*N* = 34 (*n* = 35/34)	*N* = 35 (*n* = 36/35)
Parent-report	79.73 (16.52)	82.14 (15.59)	81.67 (18.65)
Self-report	77.68 (17.31)	81.5 (20.24)	81.49 (16.77)
Agreement CCC [95% CI]	0.48 [0.19–0.69]	0.6 [0.35–0.77]	0.66 [0.42–0.80]

Age-group 14–17	*N* = 36 (*n* = 36/36)	*N* = 32 (*n* = 33/33)	*N* = 32 (*n* = 33/32)
Parent-report	72.61 (18.91)	72.27 (23.39)	77.12 (21.05)
Self-report	76.69 (16.12)	70.06 (21.14)	76.47 (18.99)
Agreement CCC [95% CI]	0.46 [0.18–0.68]	0.63 [0.38–0.7]	0.64 [0.37–0.8]

VAS by sex			
Boys	*N* = 31 (*n* = 31/31)	*N* = 30 (*n* = 31/30)	*N* = 29 (*n* = 30/29)
Parent-report	75.68 (18.57)	78.03 (18.6)	78.17 (22.52)
Self-report	77.42 (16.74)	77.07 (24.75)	79.72 (20.94)
Agreement CCC [95% CI]	0.47 [0.14–0.7]	0.62 [0.37–0.78]	0.76 [0.56–0.88]

Girls	*N* = 42 (42/42)	*N* = 36 (*n* = 37/37)	*N* = 38 (*n* = 38/38)
Parent-report	76.62 (17.74)	76.78 (21.74)	80.51 (17.7)
Self-report	77.02 (16.75)	74.89 (18.39)	78.61 (15.47)
Agreement CCC [95% CI]	0.48 [0.21–0.68]	0.66 [0.44–0.81]	0.5 [0.23–0.7]

Overall VAS T1: 73 complete dyads. T2: 68 parents and 67 children, 66 complete parent–child dyads. T3: 69 parents and 67 children, 67 complete parent–child dyads

VAS by age-group: T1. 10–13 years: 37 complete dyads. 14–17 years: 36 complete dyads. T2: 10–13 years: Parent-report *n* = 35, 34 self-report, 34 complete parent–child dyads. 14–17 years: Parent-report *n* = 33, 32 self-report, 32 complete parent–child dyads. T3. 10–13 years: Parent-reports *n* = 36, 35 self-report, 35 complete parent–child dyads. 14–17 years: Parent-reports *n* = 33, 32 self-report, 32 complete parent–child dyads

VAS by sex: T1. Boys—31 complete dyads, girls 42 complete dyads. T2: Parent-report boys *n* = 31, 30 boys self-report, 30 complete parent-boy dyads. Parent-reports girls *n* = 37, 37 girls self-report, 36 complete parent-girl dyads. T3: Parent-reports boys *n* = 30, 29 boys self-report, 29 complete parent-boy dyads. Parent-reports girls *n* = 39, 38 girls self-report, 38 complete parent-girl dyads

CCC: concordance correlation coefficient. Altman’s scale interpretation: < 0.2 = poor, 0.21–0.4 = fair, 0.41–0.6 = moderate, 0.61–0.8 = good, 0.81–1 = very good

### Changes in EQ-5D-Y-3L VAS, and reliable clinical change in EF

After collapsing the calculated BRI “no clinical change” and “negative clinical change” groups into one, neither the “no change/negative clinical change”—group nor the “positive clinical change”—group demonstrated significant improvements in VAS from T1 to T3 (*F* = 2.19, *p* = 0.12) (Table 4). No main effect of group affiliation was found (*F* = 0.29, *p* = 0.09), and there was no time*group interaction (*F* = 0.16, *p* = 0.21). For the comparable two MI-calculated clinical change groups, there was no significant improvement of the VAS over time for either of the “no change/negative clinical change” and “positive clinical change”- groups (*F* = 2.23, *p* = 0.12). A significant main effect by group affiliation was found (*F* = 4.02, *p* = *,*049), but no time*group interaction (*F* = 0.41, *p* = 0.66).

**Table 4:** Changes in HRQOL

Measure	Group	Baseline	8-week	6-months	Group	Time	Group *time	Group *time
*Mean* [95%CI]		T1	T2	T3	*p*	*p*	*p*	df
VAS BRIEF-BRI (parent-report)					0.91	0.12	0.21	1.61
	NC	74.4 [68.95–79.85]	75.2 [69.11–81.3]	75.29 [69.54–81.05]				
	CC	79.44 [72.13–86.75]	80.74 [72.44–89.03]	86.52 [78.85–94.19]				
	Total	76.92 [72.36–81.48]	77.97 [72.82–83.12]	80.9 [76.13–85.7]				
VAS BRIEF-MI (parent-report)					0.049*	0.11	0.68	0.41
	NC	73.24 [67.6–78.89]	73.49 [67.28–79.7]	76.37 [70.35–82.38]				
	CC	79.68 [72.85–86.5]	83.64 [75.89–91.39]	84.84 [77.48–92.19]				
	Total	76.46 [72.03–80.89]	78.57 [73.6–83.53]	80.6 [75.85–85.35]				

RCI group: NC = No clinical change, CC = Clinical change. Based upon the EF measures derived from the BRIEF parent-report, 41 (BRI) and 32 (MI) participants had no change RCI, 4 (BRI) and 9 (MI) participants had a negative RCI, and 25 (BRI) and 28 (MI) participants had a positive RCI. *: sig. < 0.05. BRIEF-MI 5 missing, BRIEF-BRI 4 missing

## Discussion

The present study aimed to explore the characteristic quality of health profiles across the five health states of EQ-5D-Y-3L for children and adolescents who undergo cognitive rehabilitation as perceived by both youths and their parents, and to investigate whether reliable clinical change in EF following cognitive rehabilitation is linked to perceived improvement in HRQOL. Despite our study not finding a significant change in HRQOL following the interventions, attention should be drawn towards the tendency of a differentiated association between HRQOL and distinct behavioural and metacognitive aspects of EF. Moreover, the HRQOL profile formed by the health dimensions provides important information considering how the participants appear to have only minor problems across HRQOL dimensions, and seemingly evaluate their overall health as being relatively good.

There was no significant improvement in VAS from baseline to 6-month follow-up in either of the two RCI (no/negative vs. positive clinical change) groups. This was the case for both BRI and MI, which was different to what we had expected, although a positive trend was more noticeable in the positive RCI groups. Potentially, these results could be attributed to our choice of measure in terms of it not being sensitive enough to detect significant changes that are clinically relevant in pABI. The choice of using EQ-5D-Y-3L as our generic measure was a pragmatic decision in terms of it being a good patient-oriented questionnaire frequently used in health economics research, enabling us to compare our results to others if deemed appropriate. Disease-specific measures that include items that are considered likely to be affected by the specific condition or treatment in question could be more suitable for the evaluation of clinical trials and their influence on QOL [43]. Nonetheless, the calculated RCIs of the metacognitive facet of EF (MI) stands out in terms of those reporting a positive clinical change, showing a significantly higher VAS compared to those not reporting a clinical change on metacognition already at baseline and throughout the intervention. One interpretation could be how the metacognitive aspect of EF plays a key role in predicting participation and regulation of activities or vice versa, subsequently contributing to the corresponding physical components within HRQOL [44]. One of the dimensions reported as being most problematic was that of feeling pain and/or discomfort. We venture this to be a possible indicator of the EQ-5D-Y-3L capability to capture an important nuance of the association between HRQOL and the metacognitive aspect of EF.

Some may question the relevance of applying RCI (clinical change) as grouping variables rather than differentiating by intervention group (in this case pGMT vs. pBHW). The intervention groups have been similar without any significant differences on any of the primary and secondary outcomes. We argue that for the present study in which the purpose was to investigate HRQOL as perceived by the participants, RCI is a means to provide us with information that would have been lost otherwise. For instance, it is interesting to note that even though not significant, those who had a positive clinical change in EF following the intervention, had higher VAS already at baseline and throughout the intervention. Whether this is an expression of the children being more satisfied with their HRQOL because of their capabilities in EF [18], or if it rather demonstrates how children reporting poorer HRQOL are struggling with specific aspects of EF remains to be decided. Whatever the direction, HRQOL could be a more reliable point of reference for predicting treatment outcome than neuropsychological assessments. Compared to adult ABI, the extent and pervasiveness of problems are less likely to be identified until years after the acute period and initial recovery [45], as deficits may not become fully apparent until the cognitive processes are expected to be fully developed [46], or until the cognitive and social demands and expectations to the child reach a critical level [8]. Many of these children will perform within the normal cognitive range, yet they undeniably can display problems with EF, resulting in impaired adaptive and social functioning [47]. This illustrates how determining a child’s functioning level or even treatment outcome solely on cognitive measures can have its limitations in this clinical population. How treatment and outcome is perceived by both the child and their family, might offer a better way to evaluate the treatment’s effectiveness and determine whether a neurocognitive intervention should be implemented any further. Considering the association between EF and HRQOL, improvements in EF could potentially improve HRQOL accordingly. Hence, if we rethink how the success of a neurocognitive interventions that targets EF should be measured, more children could benefit.

Our results corroborate the important role cognition has to life quality. Specifically, our study suggests that improvement in metacognitive rather than the behavioural regulation aspect of EF is likely to positively influence HRQOL. However, as our sample was within the clinically normal cognitive range according to BRIEF, their cognitive problems in some areas could be too subtle to be detected by the EQ-5D-Y-3L. A 3-level value system for reported problems on the HRQOL dimensions may not provide a good enough differentiation of perceived problems, resulting in, on a day-to-day basis, the children’s performance therefore being perceived as acceptable enough for them to pass as relatively well-functioning. Changes in behavioural regulative processes could potentially be obscured. It is noteworthy that in adult ABI, facets to EF like inhibition, emotion control, and self-monitoring are found to contribute quite strongly to the mental components of HRQOL [44]. Considering the second most problematic dimension reported on the EQ-5D-Y-3L was that of “feeling worried, sad, or unhappy”, this is clearly an important domain to investigate further. A body of research demonstrates that there are characteristic cognitive growth curves for various EF subdomains that are seemingly in play in pABI, signifying the importance of time when interpreting level of function and disability in this clinical population [9, 48, 49]. In any case, these children and adolescents may have reduced metacognitive skills and therefore have limited insight into the impact of their reduced executive skills on their performance [50]. Additionally, how parents and children report HRQOL are most likely influenced both by measure used and the age of the child [51]. In the end, however, whether a change in HRQOL would be defined as clinically meaningful is set by the premise of who’s experiencing it [52].

Regarding the estimation of HRQOL, very few participants reported the worst severity level (level 3) on the various EQ-5D-Y-3L dimensions. The health profile of 11111 was for both the parent- and self-reports the most common profile at all time points. Notably, “feeling pain and discomfort”, and “being worried”, seem to be the dimensions that are most problematic as reported by both the youths and parents. Potentially we could have obtained a more nuanced picture of their health profiles if we had chosen the 5-level version of the EQ-5D-Y. However, as mainly the first and second levels were used by most participants, it is not unreasonable to believe that 3 levels are sufficient. Moreover, in a systematic review, a similar trend was noted, especially in the general population of school pupils, but also among some patient populations [33]. The authors highlight that this skewness is not unexpected as the EQ-5D is constructed to measure deviations in health, rather than positive health. Arguably, extending the number of levels does not necessarily give clear superiority to the EQ-5D-Y-5L over EQ-5D-Y-3L. In fact, relevant to our study, the selection of EQ-5D-Y-3L may be more suitable than the 5-level, when the population of the study is younger or where a lower literacy level is anticipated [53].

Parents and youths were mostly in agreement when estimating their HRQOL. Notably, there was a moderate discrepancy in child-parent reports on the two health dimensions “feeling pain and discomfort”, and “being worried”. Over time, the disagreement was reduced for the overall group. The problem of proxy-child disagreement is well-known across various populations. Children and parent ratings of HRQOL dimensions that are less observable (e.g., emotional and social functioning) tend to be in less agreement than for dimensions related to e.g., physical functioning and participation in general [23, 54–56]. Specifically, in pABI populations, studies have found evidence for the parents to report less favourable outcomes on HRQOL [14]. Other studies find parents to rate the HRQOL of their children higher than the children do themselves across HRQOL dimensions [18, 19]. Indeed, when exploring HRQOL of young individuals in the chronic phase of pABI, parent-reports could be sufficient when looking at the overall clinical group. However, we know children with pABI do tend to report a broader range of negative emotions such as worry, sadness, shame, and guilt as being much more problematic than physical limitations [57]. How much children and parents focus on psychosocial consequences compared to physical concerns in relation to having a chronic health condition may be influenced by the severity of injury symptoms and how it is perceived [3, 58]. Thus, parents may not be able to fully understand the experiences of their children. This has clinical relevance considering parents, based upon their perceptions of their children’s health, largely will function as the clinical decision-makers [55].

### Strengths and limitations

The present study represents an important contribution to the knowledge base on children and adolescents in the chronic phase of pABI regarding their HRQOL. Given the robust RCT design targeted to improve EF, a factor that is well-known to influence HRQOL, preliminary findings are provided on how a cognitive intervention that is specially designed for this clinical group potentially can be used to influence HRQOL. Our findings must be interpreted with some caution, however, due to methodological limitations.

Our study is limited to Norwegian children and adolescents with pABI, which restricts us to a small and heterogeneous study sample. Studies with large enough samples so differentiation of various pABI aetiologies can be performed would be preferable considering the heterogeneity of this clinical group. However, the present study is unique regarding the clinical population that is chosen for study, and the choice of EQ-5D-Y-3L as the measurement. The various EQ-5D measures are still being validated for use in Norway, and consequently, there is yet not a social value set available for the EQ-5D-Y-3L/5L. However, the measure has been well-validated and is reliable in other populations, thus, studies like ours are therefore valuable and needed.

Considering our data is collected in tandem with an RCT, another relevant issue emerges that is linked to our choice of the EQ-5D-Y-3L as our HRQOL measure. As a generic measure, it is not necessarily the most favourable choice when evaluating our RCT, as such measures tend to lack sensitivity to detect small but clinically significant changes in HRQOL over time [43]. EQ-5D questionnaires are more often thought used in health economics appraisals, but its usefulness in non-economic contexts such as measuring HRQOL is not uncommon [59]. In the circumstance of our study, both intervention groups were designed according to the assumption that if operating within a framework that focuses on real-life contexts including everyday routines of the child’s life at home, this will generate a potential generalisable effect on other areas of functioning that is important for life quality [35, 60]. We believe that our choice of measure can serve both purposes, at least it can set a discourse on this matter.

Lastly, the significant physical, cognitive, emotional, and social changes that children undergo during development represent an important methodological challenge when investigating HRQOL among young children and adolescents. The changes in their priorities and their value system to how they rate various life domains will be substantial and noticeable, and thus, may impact their reporting of HRQOL at different time points [10]. Hence, assessment of HRQOL of all children with pABI should be an inherent element of their health monitoring from the time the pABI occurs and into adulthood.

## Supplementary Information

Below is the link to the electronic supplementary material.

Supplementary file1 (DOCX 38 KB)

Supplementary file2 (DOCX 15 KB)

## Data Availability

The data presented in this study will not be widely accessible due to Norwegian legislation and for the protection of personal information of vulnerable young children and adolescents. Further inquiries can be directed to the corresponding author.
