# Supplementary material for: Health-related quality of life in children and adolescents with paediatric acquired brain injury: Secondary data analysis from a randomised controlled trial
Source: Qual Life Res. 2024 Nov 22;34(2):577–88. doi: 10.1007/s11136-024-03838-2 (PMC11865218; doi:10.1007/s11136-024-03838-2)
Supplement: Supplementary file 1 — Supplementary file1 (DOCX 38 KB) [file 11136_2024_3838_MOESM1_ESM.docx]

**37** completed post-intervention assessment (T2)

**36** completed post-intervention assessment (T2)

**38** Allocated active control group paediatric Brain health workshop

**37** completed allocated treatment

1 did not complete allocation

1 excluded based on functional level contrary to participation

**38** Allocated experimental group paediatric Goal Management Training

**36** completed allocated treatment

2 did not complete allocation

1 drop-out after session #2

1 excluded based on functional level contrary to participation

**38** assessed at baseline (T1)

**38** assessed at baseline (T1)

**12** Excluded in screening interview

**2** Declined to participate

**10** Did not meet inclusion criteria

**9** Insufficient EF complaints

**1** Excluded based on function

**87 Randomised**

**11** Pre-inclusion attrition (after randomisation, before T1) ^a^

**9** withdrew from participation

**2** excluded based on function

**99** Eligible for screening

**770** Identified

**223** Invited based on discharge diagnosis and medical record information

**124** Excluded in first telephone contact

**56** Declined to participate

**46** Did not meet inclusion criteria

**36** Insufficient EF complaints

**7** Excluded based on function

**3** Other

**22** other reasons

**15** no contact

**7** wrongly invited

**36** at 6 months follow-up (T3)

1 lost to follow-up (cancer recurrence)

**35** at 6 months follow-up (T3)

1 lost to follow-up (cancer recurrence)

Supplementary Figure S1. Consort flow diagram. ^a^ Nine withdrew while waiting for start of assigned intervention group (worsening of illness, medication testing, intensifying physical rehabilitation), 2 were excluded post-randomisation (before baseline) after identification of violations of eligibility criteria not previously communicated.
