# Supplementary material for: Health-related quality of life in children and adolescents with paediatric acquired brain injury: Secondary data analysis from a randomised controlled trial
Source: Qual Life Res. 2024 Nov 22;34(2):577–88. doi: 10.1007/s11136-024-03838-2 (PMC11865218; doi:10.1007/s11136-024-03838-2)
Supplement: Supplementary file 2 — Supplementary file2 (DOCX 15 KB) [file 11136_2024_3838_MOESM2_ESM.docx]

**Table S1. EQ-5D-Y-3L health dimensions, severity category response for parent- and self-report**

|  | | Responses *n* (%) total sample | | | | | |
| --- | --- | --- | --- | --- | --- | --- | --- |
| EQ-5D-Y dimension | | Baseline (T1) | | 8 weeks (T2) | | 6-months (T3) | |
|  | | Parent | Self | Parent | Self | Parent | Self |
| Mobility | No problems | 60 (82.2) | 59 (80.8) | 57 (78.1) | 55 (75.3) | 55 (75.3) | 56 (76.7) |
|  | Some problems | 11 (15.1) | 13 (17.8) | 10 (13.7) | 10 (13.7) | 12 (16.4) | 10 (13.7) |
|  | A lot of problems | 2 (2.7) | 1 (1.4) | 2 (2.7) | 2 (2.7) | 2 (2.7) | 1 (1.4) |
| Looking after myself | No problems | 62 (84.9) | 66 (90.4) | 59 (80.8) | 64 (87.7) | 60 (82.2) | 63 (86.3) |
|  | Some problems | 8 (11.0) | 5 (6.8) | 9 (12.3) | 2 (2.7) | 8 (11.0) | 3 (4.1) |
|  | A lot of problems | 3 (4.1) | 2 (2.7) | 1 (1.4) | 1 (1.4) | 1 (1.4) | 1 (1.4) |
| Doing usual activities | No problems | 39 (53.4) | 46 (63.0) | 43 (58.9) | 42 (57.5) | 46 (63.0) | 49 (67.1) |
|  | Some problems | 25 (34.2) | 22 (30.1) | 19 (26.0) | 22 (30.1) | 15 (20.5) | 15 (20.5) |
|  | A lot of problems | 9 (12.3) | 5 (6.8) | 7 (9.6) | 3 (4.1) | 6 (8.2) | 3 (4.1) |
| Having pain or discomfort | No problems | 35 (47.8) | 31 (42.5) | 34 (46.6) | 25 (34.2) | 31 (42.5) | 31 (42.5) |
|  | Some problems | 29 (39.7) | 38 (52.1) | 27 (37.0) | 34 (46.6) | 29 (39.7) | 30 (41.1) |
|  | A lot of problems | 9 (12.3) | 4 (5.5) | 8 (11.0) | 8 (11.0) | 9 (12.3) | 6 (8.2) |
| Feeling worried, sad, or unhappy | No problems | 33 (45.2) | 41 (56.2) | 39 (52.4) | 44 (60.3) | 40 (54.8) | 44 (60.3) |
|  | Some problems | 36 (49.3) | 28 (38.4) | 25 (34.2) | 21 (28.8) | 27 (37.0) | 21 (28.8) |
|  | A lot of problems | 4 (5.5) | 3 (4.1) | 5 (6.8) | 2 (2.7) | 2 (2.7) | 0 (0.0) |

Parent-report: T2: 4 missing: Mobility, Looking after myself, Doing usual activities, Having pain or discomfort, Feeling worried, sad, or unhappy. T3: 4 missing: Mobility, Looking after myself, Having pain or discomfort, Feeling worried, sad or unhappy, 6 missing: Doing usual activities. Self-report: T1: 1 missing: Feeling worried, sad, or unhappy. T2: 6 missing: Mobility, Looking after myself, Doing usual activities, Having pain or discomfort, Feeling worried, sad or unhappy. T3 6 missing: Mobility, Looking after myself, Doing usual activities, Having pain or discomfort, 8 missing: Feeling worried, sad or unhappy.
